# GLI3 Promotes Invasion and Predicts Poor Prognosis in Colorectal Cancer

**DOI:** 10.1155/2021/8889986

**Published:** 2021-01-09

**Authors:** Mingyang Shen, Zhengyuan Zhang, Ping Wang

**Affiliations:** ^1^Department of Vascular Surgery, Huai'an First People's Hospital Affiliated to Nanjing Medical University, Huai'an, Jiangsu, China; ^2^Department of Gastrointestinal Surgery, Huai'an First People's Hospital Affiliated to Nanjing Medical University, Huai'an, Jiangsu, China

## Abstract

**Purpose:**

The epithelial–mesenchymal transition (EMT) is a key hallmark of cancer which promotes malignant progression, especially during the process of cancer invasion. A better understanding of EMT will help elucidate the molecular mechanism underlying colorectal cancer (CRC) metastasis and may provide new insights into the identification of potential biomarkers and therapeutic targets.

**Methods:**

A series of bioinformatic approaches were combined and identify GLI3 as a potential key regulator in EMT. In vitro experiments were performed to knockdown GLI3 expression in two CRC cell lines and to reveal the oncogenic role of GLI3 in CRC. qRT-PCR and western blot were performed to show the influence of GLI3 in EMT and downstream pathways. The Kaplan-Meier analysis and log-rank test were used to evaluate the prognostic value of GLI3 in CRC patients.

**Results:**

GLI3 was identified as a key regulator in coexpression and protein-protein interaction (PPI) networks involved in EMT. Bioinformatic analyses indicated that GLI3 had a high correlation with EMT markers in CRC. In vitro experiments showed that GLI3 knockdown attenuated the migratory and invasive capacities of CRC cells via influencing EMT property, especially by regulating phosphorylation of ERK signaling pathway. In addition, higher expression of GLI3 predicts worse prognosis in CRC patients.

**Conclusions:**

In summary, we presented the first evidence that GLI3 could promote the migratory and invasive capacities of CRC cells by regulating the EMT process. Our study might provide some useful clues to a better understanding of GLI3 in EMT during CRC progression.

## 1. Background

According to the latest released Global Cancer Statistics, colorectal cancer (CRC) becomes the world's third most diagnosed cancer, with an annual incidence of over 1.4 million cases [1]. Despite the recent advances in treatment [2, 3], the 5-year overall survival remains poor, with the rate about 60% [4]. Distant metastases were observed in 20% of newly diagnosed patients, and the biggest challenge is the judgment of the metastasis situation when CRC is diagnosed [5, 6]. Thus, there is an urgent need to reveal the mechanism underlying CRC metastasis, which may benefit to identification of novel diagnostic biomarkers and development of therapeutic targets [5, 7].

The epithelial-mesenchymal transition (EMT) is a key hallmark of cancer which promotes malignant progression, especially during the process of cancer invasion [8, 9]. A better understanding of EMT will help elucidate the molecular mechanism underlying CRC metastasis and may provide new insights into the personalized management of CRC patients [10]. GLI1, the first member identified of GLI family, has been widely reported and promotes malignant progression in various cancers via influencing the hedgehog signaling pathway. GLI3, another member of GLI zinc finger family, was also widely reported in various human cancers. GLI3 was significantly upregulated in solid cancer types including pancreatic cancer, cervical cancer, and oral squamous cancer [11]. In oral squamous cancer, silencing of GLI3 attenuated the stemness and invasive capacity [12]. In addition, GLI3 affects proliferation and apoptosis in cervical cancer cells and was targeted by miRNA-218 and miRNA-506 [13, 14]. These evidences suggested that GLI3 was involved in the malignant progression in different cancer types. However, the role of GLI3 in EMT remains unclear.

In this study, we identified GLI3 as a key regulator of EMT in CRC. Using a series of bioinformatic analyses, we found that GLI3 was involved in the coexpression and protein-protein interaction network of EMT process in CRC. In vitro experiments showed that silencing of GLI3 weakened the migratory and invasive capacities of CRC cells via influencing EMT by regulating the phosphorylation of ERK signaling pathway. These findings indicated that GLI3 plays an important role in CRC progression and may serve as a promising biomarker in CRC.

## 2. Materials and Methods

### 2.1. Cell Culture and Transfection

The human CRC cell lines SW480 and DLD1 were purchased from American Type Culture Collection. Cells were cultured in DMEM media supplemented with 10% fetal bovine serum (FBS) and penicillin/streptomycin with 37°C in a humidified incubator with 5% CO_2_. Transfection of small-interfering RNA (siRNA) was performed following the standard protocols as previously reported [15]. After transfection for 48 h, the knockdown efficiency was checked by qRT-PCR and western blot. Specific siRNA targeting GLI3 were designed: 5′-AAUGAGGAUGAAAGUCCUGGATT-3′ and 5′-UCCAGGACUUUCAUCCUCAUU TT-3′. As a nonspecific control siRNA, a scrambled siRNA (5′-UUCUCCGAACGUGUCACGUTT-3′, 5′-ACGUGACACGUUCGGAGAATT-3′) was used.

### 2.2. RNA Extraction and Real-Time Quantitative PCR

Total RNA was extracted from cultured cells using TRIzol reagent (Invitrogen, CA, USA) compiled with manufacturer's guidelines. 1000 ng total RNA was reversely transcribed to a final volume of 20 *μ*L using a reverse transcription kit (Takara). The qRT-PCR analysis was performed with SYBR Select Master Mix (Applied Biosystems) and QuantStudio TM 6 Flex Real-time PCR system, and the qRT-PCR reaction parameters were set as previously reported [15]. Each sample was run in triplicate, and the relative expression was calculated and normalized to GAPDH with the 2^-*ΔΔ*CT^ method. The qRT-PCR primers for GLI3, VIM, CDH2, ZEB1, and GAPDH were shown in Supplementary Table 1.

### 2.3. Protein Preparation and Western Blot

Cells were harvested and treated with lysis buffer on ice, and then, the protein was quantified with a BCA kit. The procedures of western blot were complied with standard protocols as previously reported [15]. Antibodies against GLI3 (1 : 1000, Abcam, Cat. No. ab6050), GAPDH (1 : 1000, Abcam, Cat. No. ab8245), VIM (1 : 500, Abcam, Cat. No. ab92547), ZEB1 (1 : 1000, Abcam, Cat. No. ab203829), and CDH2 (1 : 500, Abcam, Cat. No. ab202030) were used during overnight incubation. After being washed in TBS-T, membranes were incubated with goat anti-rabbit HRP-conjugated secondary antibody (1 : 10,000; Abcam) or goat anti-mouse HRP-conjugated secondary antibody (1 : 10,000; Abcam) for 2 h at room temperature. The blots were visualized by the ECL detection (Thermo Scientific). All experiments were repeated in triplicate.

### 2.4. Migration and Invasion Assays

In the Transwell assay, 20,000 transfected CRC cells were cultured in the serum-free media and then plated to the upper chamber (8-*μ*m pore size; Millipore, Billerica, MA, USA). Complete medium with 10% FBS was added to the lower chamber. After incubation of 24 h at 37°C and 5% CO_2_, CRC cells that migrated across the pore were fixed and stained and quantified by the microscopy. Migrated cells were chosen from 3 random fields, and the numbers were, respectively, counted to acquire a mean value. For the Matrigel assay, 20,000 transfected CRC cells were plated on the upper chamber with a Matrigel-coated membrane (BD Biosciences) in a serum-free medium. The lower chamber contained a complete medium with 10% FBS. After 48 h incubation, the invasive CRC cells were fixed, stained, and counted as described above.

### 2.5. Bioinformatic and Statistical Analyses

Normalized RNA-seq data and follow-up information on survival including overall survival (OS) and progression-free survival (PFS) of CRC samples were acquired from The Cancer Genome Atlas (TCGA). In addition, transcriptome data and survival information including OS and recurrence-free survival (RFS) of GSE17536 and GSE39582 were acquired from Gene Expression Omnibus (GEO). In microarray analysis, probe IDs of GLI3 were mapped to the gene symbol according to the corresponding annotation file, and expression measurements of all probes related to GLI3 were averaged to obtain a single value. The performance of EMT in each sample was quantified by a single-sample gene set enrichment analysis (ssGSEA) algorithm [16] based on transcriptome profiling data and the EMT gene signature retrieved from the Molecular Signatures Database (MSigDB) [17]. To investigate the potential pathways influenced by GLI3 in CRC, the transcriptome data of GLI3-low and GLI3-high samples from TCGA were analysed using GSEA. Weighted gene coexpression network analysis (WGCNA) [18] was used to construct a scale-free coexpression network using the R package “WGCNA” and to identify a gene module that shows the highest correlation with EMT, and CEMiTool [19] was used to visualize the networks of coexpression and protein-protein interaction (PPI) in the EMT module. Webtools GeneMANIA [20] and GSCALite [21] were used to construct the GLI3-related EMT network and pathways involved in CRC. GEPIA [22] was used to show the Pearson correlation values between GLI3 and EMT markers including CDH2, TWIST, VIM, and ZEB1. Student's *t*-test was used to analyse differences between groups in variables with a normal distribution. The Kaplan-Meier method was used to draw survival curves, and the log-rank test was performed to evaluate survival difference with the best cut-off value. The best cut-off values in each cohort were determined using a bioinformatic tool named X-tile, which is used for biomarker assessment and outcome-based cut-point optimization with the maximum statistics in survival analyses [23]. *p* value less than 0.05 was considered statistically significant.

## 3. Results

### 3.1. Identification of an EMT-Related Gene Module in CRC

**Figure 1:**
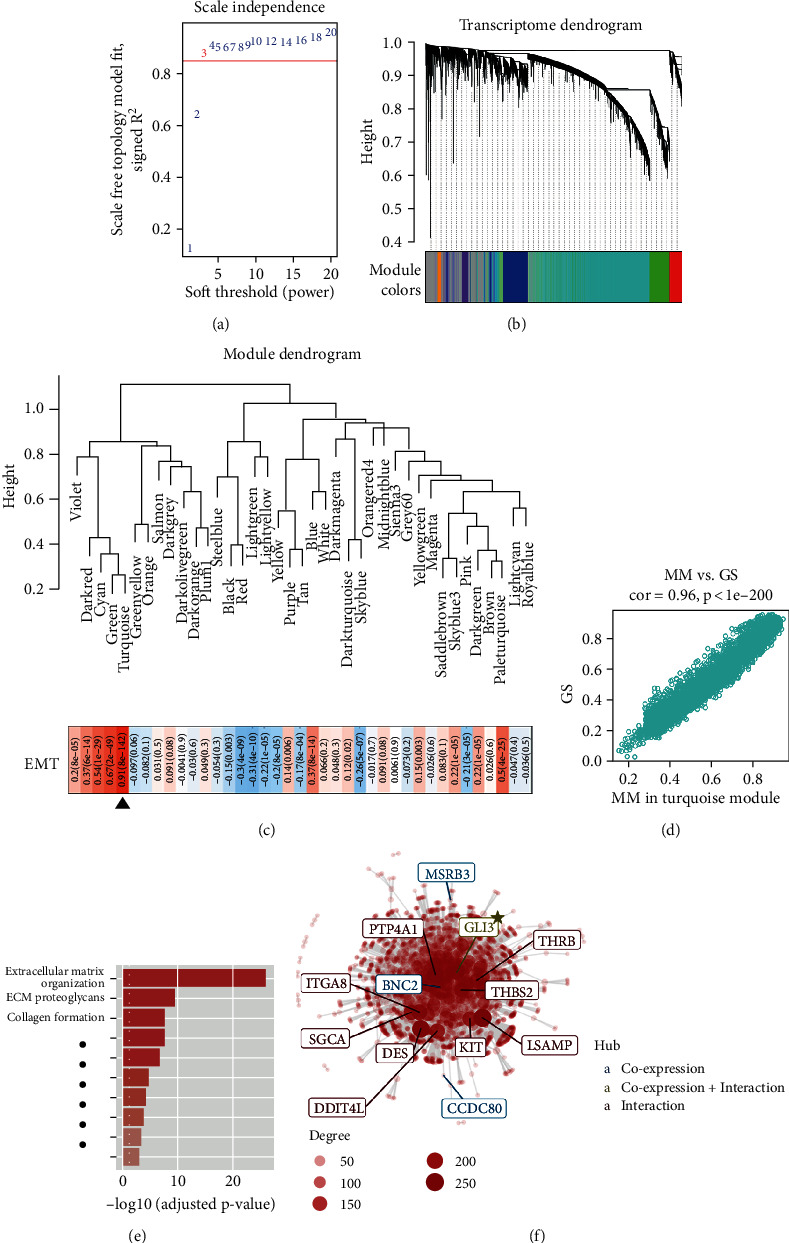
Identification of an EMT-related gene module in CRC. (a) Power of *β* = 5 was chosen as the optimal soft threshold. (b) A total of 39 gene modules were generated. (c) The turquoise module had the highest correlation (*r* = 0.91, *p* = 8*e* − 142) with EMT Z-score. (d) There is a high correlation between the module membership (MM) and gene significance (GS) in the turquoise module. (e) Reactome enrichment analysis showed that the top three pathways in the turquoise module were labelled with “Extracellular matrix organization,” “ECM proteoglycans,” and “Collagen formation.” (f) A regulation network composing coexpression genes and protein-protein interaction (PPI) was constructed, and the key regulators in the EMT module were coloured with different colours according to the roles.

WGCNA was performed with transcriptome profiling data and EMT Z-scores to construct a scale-free coexpression network. A power of *β* = 5 was chosen as the optimal soft threshold (Figure 1(a)). In the transcriptome clustering dendrogram shown in Figure 1(b), a total of 39 gene modules were generated, and we observed that the turquoise module had the highest correlation (*r* = 0.91, *p* = 8*e* − 142) with EMT Z-score (Figure 1(c)). Thus, we considered the turquoise module as the “EMT module.” In addition, as shown in Figure 1(d), there is a highly positive correlation between the module membership (MM) and gene significance (GS) in the turquoise module, indicating the high consistency in the coexpression module. Reactome enrichment analysis showed that the top three pathways in the turquoise module were labelled with “Extracellular matrix organization,” “ECM proteoglycans,” and “Collagen formation,” which were involved in the invasive process (Figure 1(e)). A regulation network composing coexpression genes and protein-protein interaction (PPI) was constructed using CEMiTool, and the key regulators in the EMT module were coloured with different colours according to the roles (Figure 1(f)).

### 3.2. GLI3 Is a Critical Regulator in EMT

**Figure 2:**
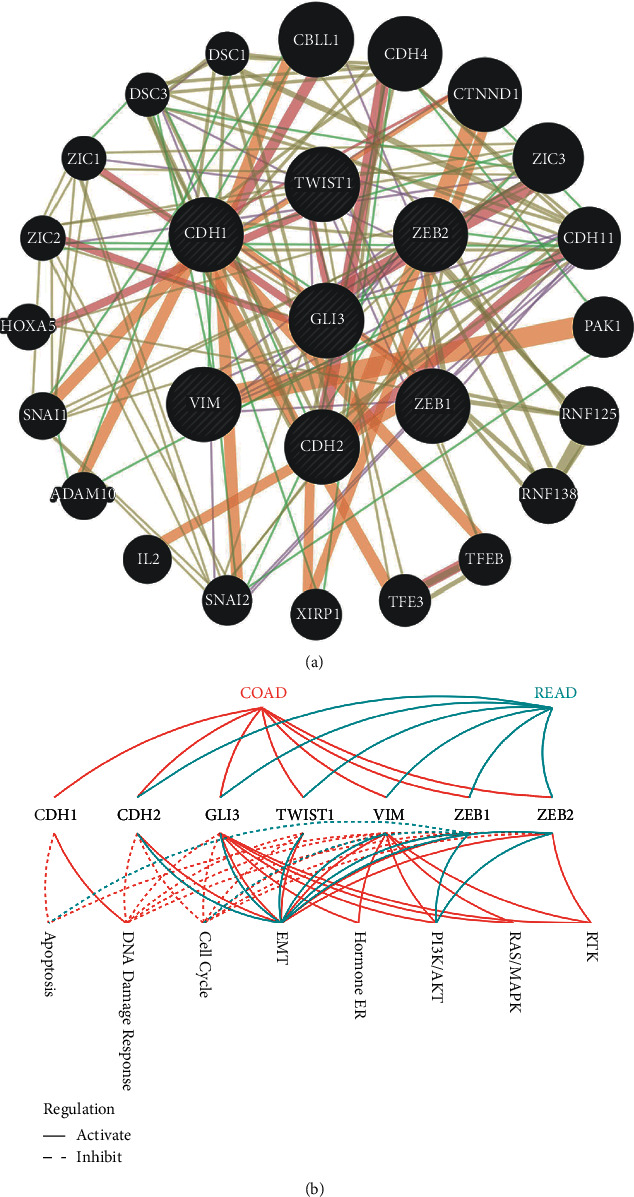
GLI3 is a critical regulator in EMT. (a) GeneMANIA was used to analyse the correlation and interaction between GLI3 and other EMT markers. (b) GSCALite analysed the correlations between those EMT markers and different pathways in both colon adenocarcinoma (COAD) and rectal adenocarcinoma (READ).

As shown in Figure 1(f), GLI3 acted as a core regulator in both coexpression network and PPI network, which suggests GLI may be a critical regulator in EMT process. Then, a web tool GeneMANIA was used to analyse the correlation and interaction between GLI3 and other EMT markers, and we found that GLI3 was located in the core position in the EMT regulation network, and important EMT markers such as CDH1, ZEB1, and VIM had high correlations and tight interactions with GLI3 (Figure 2(a)). In addition, GSCALite analysed the correlations between those EMT markers and different pathways in both colon adenocarcinoma (COAD) and rectal adenocarcinoma (READ), and the results indicated that GLI3 had a tight link with EMT compared to other traditional EMT markers (Figure 2(b)). These findings demonstrated that GLI3 acted as a critical regulator in EMT of CRC.

### 3.3. GLI3 Promotes EMT via Regulating p-ERK Signaling Pathway

**Figure 3:**
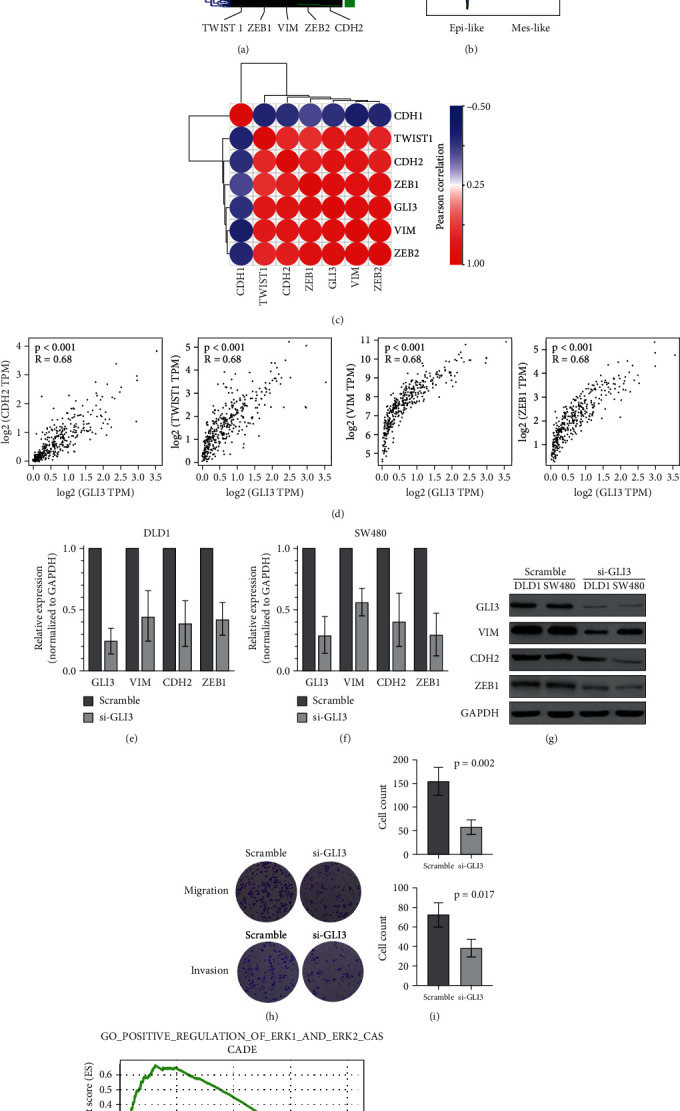
GLI3 promotes EMT via regulating p-ERK signaling pathway. (a) TCGA CRC samples were divided into two groups: epithelial-like phenotype (green) and mesenchymal-like phenotype (red). (b) GLI3 expression was significantly elevated in the mesenchymal-like group. (c) Correlation heat map indicated that GLI3 was negatively correlated with CDH1, and positively correlated with TWIST1, CDH2, ZEB1, ZEB2, and VIM. (d) Details of the correlation between logTPM-transformed GLI3 and CDH2, TWIST1, VIM, and ZEB1. (e–g) Two CRC cell lines DLD1 and SW480 were used to knockdown GLI3 expression. In both cell lines, specific siRNA targeting GLI3 had an optimal efficiency of silencing, and western blot was further performed to validate the knockdown efficiency at the protein level. qRT-PCR and western blot analyses demonstrated that the EMT markers were significantly downregulated in GLI3-silencing cells at both mRNA level and protein level. (h, i) Silencing of GLI3 greatly attenuated both the migratory and invasive capacities of DLD1 and SW480 cells in response to siRNA-treatment. (j) GSEA analysis indicated that high expression of GLI3 is involved in the function of positive regulation of ERK1/2 cascade. (k) Western blot demonstrated that the pERK1/2 activation was significantly attenuated in GLI3-knockdown groups.

The expression matrix of five important EMT markers including TWIST1, ZEB1, VIM, ZEB2, and CDH2 in CRC from TCGA was used to perform the hierarchical clustering analysis to divide the TCGA CRC samples into two groups: epithelial-like phenotype (green) and mesenchymal-like phenotype (red) (Figure 3(a)). And we observed that GLI3 expression was significantly elevated in the mesenchymal-like group (Figure 3(b)). Correlation heat map in Figure 3(c) indicated that GLI3 was negatively correlated with CDH1, and positively correlated with TWIST1, CDH2, ZEB1, ZEB2, and VIM (Figure 3(c)). The details of the correlation between logTPM-transformed GLI3 and CDH2, TWIST1, VIM, and ZEB1 were shown in Figure 3(d), and R in each panel represents the Pearson correlation value. Then, two CRC cell lines DLD1 and SW480 were used to knockdown GLI3 expression and perform further study. In both cell lines, specific siRNA targeting GLI3 had an optimal efficiency of silencing, about 75% in DLD1 (Figure 3(e)) and 70% in SW480 (Figure 3(f)), and western blotting analysis was further performed to validate the silencing efficiency at the protein level (Figure 3(g)). In addition, qRT-PCR and western blotting analyses showed that the expressions of VIM, CDH2, and ZEB1 were significantly downregulated in GLI3-silencing cells at both mRNA level and protein level (Figures 3(e)–3(g)).

Migration and invasion assays were performed to investigate the influence of GLI3 knockdown in CRC cells, and we observed that silencing of GLI3 greatly attenuated both the migratory and invasive capacities of DLD1 and SW480 cells in response to siRNA-treatment compared to the cells treated with siRNA-scramble (si-scr) (Figures 3(h) and 3(i)). GSEA analysis showed that GLI3 was positively correlated with Hedgehog signaling pathway in CRC samples (Figure S1). Furthermore, high expression of GLI3 is involved in the function of positive regulation of ERK1/2 cascade (Figure 3(j)). Western blot demonstrated that the pERK1/2 activation was significantly weakened in GLI3-knockdown group compared to scramble control (Figure 3(k)).

### 3.4. Higher GLI3 Expression Predicts Worse Prognosis in CRC Patients

**Figure 4:**
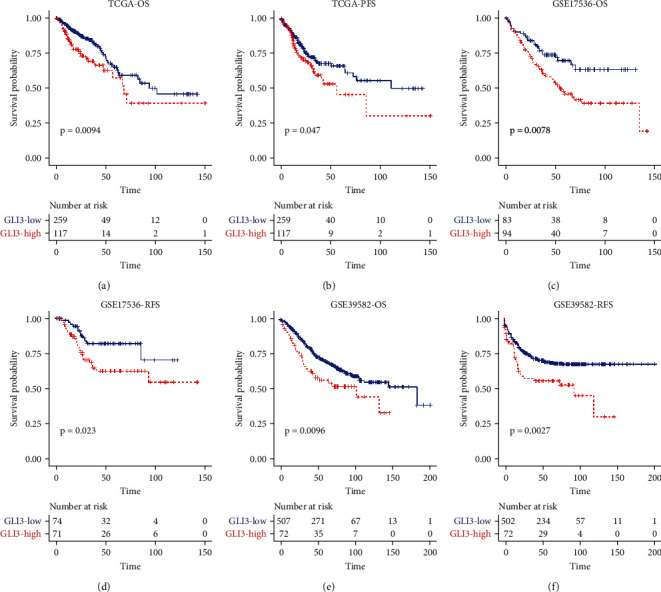
Higher GLI3 expression predicts worse prognosis in CRC patients. (a–f) CRC patients with higher expression of GLI3 exhibited worse overall survival compared to those with lower expression of GLI3 in TCGA and GEO cohorts, as well as the PFS and RFS.

To investigate the prognostic value of GLI3 in CRC, we extracted the GLI3 expression and different survival types including OS and PFS in TCGA, and OS and RFS information from two GEO datasets (GSE17536 and GSE39582). Log-rank test showed that CRC patients with higher expression of GLI3 exhibited worse overall survival compared with those with lower expression of GLI3 in TCGA and GEO cohorts, as well as the PFS and RFS (Figures 4(a)–4(f)). These findings showed that GLI3 could serve as a promising biomarker for survival prediction in CRC patients.

## 4. Discussion

Epithelial to mesenchymal transition (EMT) is a complicated biological process by which epithelial cells lose their cell polarity and cell-cell adhesion and acquire migratory and invasive capacity to become mesenchymal-like cells [24]. These changes remodel cytoskeleton and disrupt the adhesive property. It is widely acknowledged that EMT has three distinct settings, and the cancer progression-related subtype promotes normal cells to transform to malignant ones and enables primary tumor cells more invasive. An increasing number of studies have provided strong evidences that EMT plays an important role in cancer progression and metastasis in various malignancies [25]. In addition, the identification of novel oncogenes that promote the activation of EMT signaling pathway during malignant process will provide useful clues into the plasticity of tumor cells and possible potential therapeutic strategies.

In our study, we firstly quantified EMT level in CRC samples using ssGSEA method. ssGSEA is an extension of GSEA, and it calculates separate enrichment scores for each paring of a sample and gene set. Using the EMT gene set retrieved in MSigDB, the EMT level for each CRC sample was calculated. Then, different bioinformatic approaches were combined, and a coexpression and protein-protein interaction network involved in EMT was generated. We thought GLI3 as a key regulator in CRC because it functions not only in the coexpression network but also in the protein-protein interaction network. In the mesenchymal-like CRC samples, GLI3 was significantly elevated compared to the expression level in the epithelia-like samples. Furthermore, in vitro experiments were performed to reveal the biological role of GLI3 in CRC cells. Silencing of GLI3 could attenuate the migratory and invasive capacities in CRC cells, via influencing the phosphorylation of ERK signaling pathway. In addition, GSEA analysis showed that GLI3 was also positively correlated with Hedgehog signaling pathway, and these findings indicated that ERK1/2 pathway might be a downstream effect of GLI3-Hedgehog signaling cascade.

GLI1 was the most widely reported member involved in the GLI zinc finger family, and it is well acknowledged that GLI1 promotes malignant progression in cancers by regulating the Hedgehog signaling pathway [26]. Recently, accumulating studies have demonstrated that another GLI family member GLI3 was significantly upregulated in many human cancers. In oral squamous cancer, GLI3 knockdown decreases the stemness and cell invasion [12]. GLI3 affects proliferation and apoptosis in cervical cancer cells and was targeted by miRNA-218 and miRNA-506 [13, 14]. These evidences demonstrated that GLI3 was involved in the regulation of malignant process in different cancer types. However, the role of GLI3 in EMT process has never been reported. Using bioinformatic analysis, we identified GLI3 as a potential EMT regulator, and the regulation role and downstream pathway of GLI3 in EMT was subsequently investigated.

EMT is a key hallmark of cancer, especially during the process of cancer invasion and metastasis [27]. A better understanding of EMT and identification of novel oncogenes involved in EMT process will provide useful clues to the development of novel diagnostic biomarkers and therapeutic targets. As a transcription factor, GLI3 might also affect the transcription of other oncogenes and regulate the gene network involved in the CRC progression, as well as ZEB1 and TWIST1. Based on these findings in this study, we would like to explore the transcription-promoting role of GLI3 in CRC in the future work.

## 5. Conclusions

In summary, we presented the first evidence that GLI3 could enhance the migratory and invasive capacities of CRC cells by regulating the EMT process. In addition, silencing of GLI3 could affect the phosphorylation of ERK signaling pathway. Our study might provide some useful insights into a better understanding of GLI3 in EMT in CRC progression.

## Data Availability

All the available RNA-seq and microarray data and survival information on CRC were obtained from The Cancer Genome Atlas (https://portal.gdc.cancer.gov/) and Gene Expression Omnibus (https://www.ncbi.nlm.nih.gov/geo/).

## Supplementary Materials

Supplementary Materials Table S1: the sequences of qRT-PCR primers for GLI3, VIM, CDH2, ZEB1 and GAPDH. Figure S1: GSEA analysis showed that GLI3 was positively correlated with Hedgehog signaling pathway in CRC samples.

## References

[1] Siegel R. L., Miller K. D., Jemal A. (2018). Cancer statistics, 2019. *CA: A Cancer Journal for Clinicians*.

[2] Ducreux M., Chamseddine A., Laurent-Puig P. (2019). Molecular targeted therapy ofBRAF-mutant colorectal cancer. *Therapeutic Advances in Medical Oncology*.

[3] van der Jeught K., Xu H.-C., Li Y.-J., Lu X.-B., Ji G. (2018). Drug resistance and new therapies in colorectal cancer. *World Journal of Gastroenterology*.

[4] Siegel R. L., Miller K. D., Goding Sauer A. (2020). Colorectal cancer statistics, 2020. *CA: a Cancer Journal for Clinicians*.

[5] Mitani S., Taniguchi H., Sugiyama K. (2019). The impact of the Glasgow Prognostic Score on survival in second-line chemotherapy for metastatic colorectal cancer patients with BRAF V600E mutation. *Therapeutic Advances in Medical Oncology*.

[6] Loree J. M., Kopetz S. (2017). Recent developments in the treatment of metastatic colorectal cancer. *Therapeutic Advances in Medical Oncology*.

[7] Marcucci F., Stassi G., De Maria R. (2016). Epithelial-mesenchymal transition: a new target in anticancer drug discovery. *Nature Reviews. Drug Discovery*.

[8] Nieto M. A., Huang R. Y.-J., Jackson R. A., Thiery J. P. (2016). Emt: 2016. *Cell*.

[9] Zhaojie L., Yuchen L., Miao C. (2019). Gelsolin-like actin-capping protein has prognostic value and promotes tumorigenesis and epithelial-mesenchymal transition via the Hippo signaling pathway in human bladder cancer. ***Therapeutic Advances in Medical Oncology***.

[10] Vu T., Datta P. (2017). Regulation of EMT in colorectal cancer: a culprit in metastasis. *Cancers*.

[11] Matissek S. J., Elsawa S. F. (2020). GLI3: a mediator of genetic diseases, development and cancer. *Cell Communication and Signaling: CCS*.

[12] Rodrigues M., Miguita L., De Andrade N. P. (2018). GLI3 knockdown decreases stemness, cell proliferation and invasion in oral squamous cell carcinoma. *International Journal of Oncology*.

[13] Zhang J., Li S., Li Y., Liu H., Zhang Y., Zhang Q. (2018). miRNA-218 regulates the proliferation and apoptosis of cervical cancer cells via targeting Gli3. *Experimental and Therapeutic Medicine*.

[14] Wen S.-Y., Lin Y., Yu Y.-Q. (2015). miR-506 acts as a tumor suppressor by directly targeting the hedgehog pathway transcription factor Gli3 in human cervical cancer. *Oncogene*.

[15] Zhang Z., Shen M., Zhou G. (2018). Upregulation of CDCA5 promotes gastric cancer malignant progression via influencing cyclin E1. *Biochemical and Biophysical Research Communications*.

[16] Hanzelmann S., Castelo R., Guinney J. (2013). GSVA: gene set variation analysis for microarray and RNA-seq data. *BMC Bioinformatics*.

[17] Liberzon A., Birger C., Thorvaldsdottir H., Ghandi M., Mesirov J. P., Tamayo P. (2015). The molecular signatures database hallmark gene set collection. *Cell Systems*.

[18] Langfelder P., Horvath S. (2008). WGCNA: an R package for weighted correlation network analysis. *BMC Bioinformatics*.

[19] Russo P. S. T., Ferreira G. R., Cardozo L. E. (2018). CEMiTool: a Bioconductor package for performing comprehensive modular co-expression analyses. *BMC Bioinformatics*.

[20] Warde-Farley D., Donaldson S. L., Comes O. (2010). The GeneMANIA prediction server: biological network integration for gene prioritization and predicting gene function. *Nucleic Acids Research*.

[21] Liu C. J., Hu F. F., Xia M. X., Han L., Zhang Q., Guo A. Y. (2018). GSCALite: a web server for gene set cancer analysis. *Bioinformatics*.

[22] Tang Z., Li C., Kang B., Gao G., Li C., Zhang Z. (2017). GEPIA: a web server for cancer and normal gene expression profiling and interactive analyses. *Nucleic Acids Research*.

[23] Camp R. L., Dolled-Filhart M., Rimm D. L. (2004). X-tile: a new bio-informatics tool for biomarker assessment and outcome-based cut-point optimization. *Clinical Cancer Research*.

[24] Kalluri R., Weinberg R. A. (2009). The basics of epithelial-mesenchymal transition. *The Journal of Clinical Investigation*.

[25] Li J.-M., Tseng C.-W., Lin C.-C. (2018). Upregulation of LGALS1 is associated with oral cancer metastasis. *Therapeutic Advances in Medical Oncology*.

[26] Carpenter R. L., Lo H. W. (2012). Hedgehog pathway and GLI1 isoforms in human cancer. *Discovery Medicine*.

[27] Ribatti D., Tamma R., Annese T. (2020). Epithelial-mesenchymal transition in cancer: a historical overview. *Translational Oncology*.

