# Supplementary material for: GLI3 Promotes Invasion and Predicts Poor Prognosis in Colorectal Cancer
Source: Biomed Res Int. 2021 Jan 9;2021:8889986. doi: 10.1155/2021/8889986 (PMC7814942; doi:10.1155/2021/8889986)
Supplement: Supplementary Materials — Table S1: the sequences of qRT-PCR primers for GLI3, VIM, CDH2, ZEB1 and GAPDH. Figure S1: GSEA analysis showed that GLI3 was positively correlated with Hedgehog signaling pathway in CRC samples. [file 8889986.f1.docx]

Table S1: The qRT-PCR primers

| Primers | sense (5' - 3') | antisense (5' - 3') |
| --- | --- | --- |
| GLI3 | GGCCATCCACATGGAATATC | TGAAGAGCTGCTACGGGAAT |
| CDH2 | GGTGGAGGAGAAGAAGACCAG | GGCATCAGGCTCCACAGTAT |
| VIM | TGCGCCAGCAGTATGAAA | GCCTCAGAGAGGTCAGCAAA |
| ZEB1 | TGAGCACACAGGTAAGAGGCC | GGCTTTTCCCCAGAGTGCA |
| GAPDH | ACCACAGTCCATGCCATCACT | GTCCACCACCCTGTTGCTGTA |


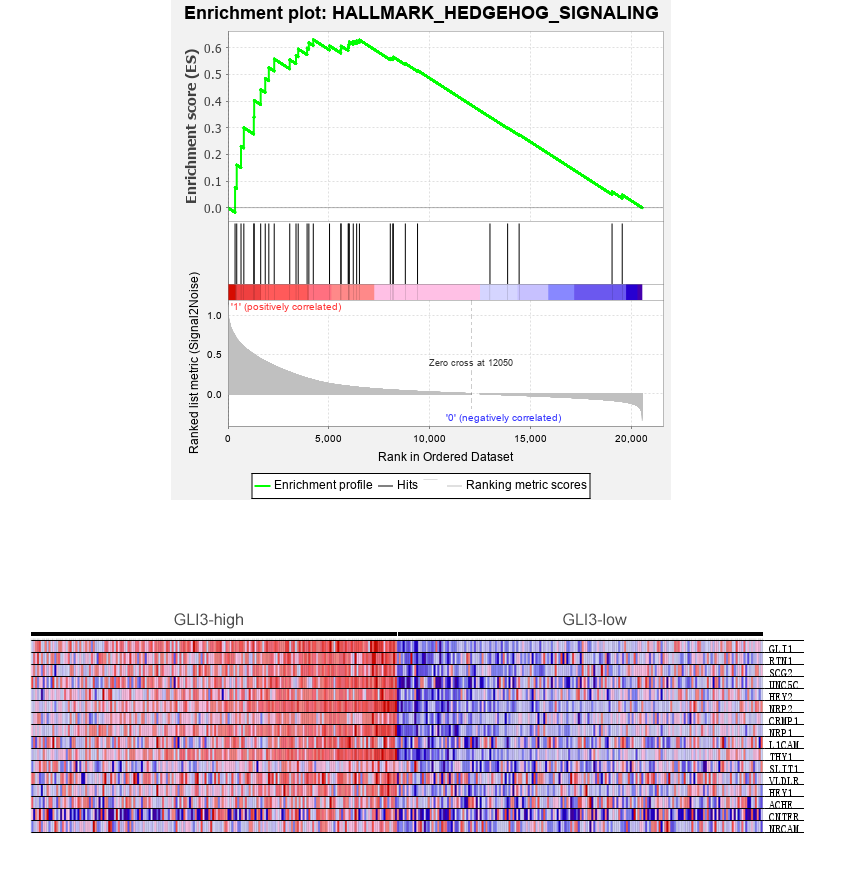


Figure S1: GSEA analysis indicated that GLI3 was positively correlated with Hedgehog signaling pathway in CRC samples.
